# Spectrum of Chest Dual-Energy Computed Tomography Findings in COVID Patients in North India

**DOI:** 10.7759/cureus.12489

**Published:** 2021-01-04

**Authors:** Sachin Khanduri, Harleen Chawla, Asif Khan, Iffat Ali, Anvit Krishnam, Saif Malik, Nazia Khan, Yunus D Patel, Surbhi ., Mufidur Rehman

**Affiliations:** 1 Radiology, Era's Lucknow Medical College and Hospital, Lucknow, IND

**Keywords:** dect, sars-cov-2, rtpcr, covid 19

## Abstract

Purpose

To study the spectrum of chest dual-energy computed tomography (DECT) imaging findings in severe acute respiratory syndrome coronavirus 2 (SARS-COV-2) or COVID-19 infected Indian patients and classify them on the basis of the Radiological Society of North America CT classification.

Method

A total of 110 reverse transcription-polymerase chain reaction (RT-PCR)-positive patients (subjects) in which noncontrast chest DECT was done in our COVID-19 care center (CCC) were enrolled in this study. The prevalence of various abnormalities of lung parenchyma due to SARS-COV-2 and their distribution with extent was recorded. Various types of lung parenchyma abnormalities due to COVID-19 were evaluated in all patients. Data were analyzed and various prevalent abnormalities were calculated as a percentage for each type. All the cases were also sorted into four major groups on the basis of the Radiological Society of North America CT classification of COVID patients.

Result

Among the total 110 patients that were enrolled in this study, 80 (72.7%) were males and 30 (27.3%) were females with a mean age of 40.5 ± 7 years (range 24-84). Out of this, we observed that 59 (53.6%) cases had abnormalities of lung parenchyma and were designated as DECT positive, whereas 51 (46.3%) cases had completely normal DECT. Only 14 (12.7%) of the patients (cases) presented with dyspnoea, 10 (9%) had hyperpnoea, whereas 12 (10.8%) had other associated comorbidities. Among the patients having abnormal DECT findings, multilobar (86%), bilateral lung field involvement (72.8%) with the ascendancy of peripheral and posterior distribution was most commonly noted. With respect to the different types of opacities noted in various patients, we found that ground-glass opacity (GGO) was the common abnormality found in almost all cases for the greatest part. Pure GGO was reported in 16 (28%), GGO admixed with a crazy-paving pattern were elicited in 17 (28.8%) and GGO mixed with consolidation was noted in 25 (42.3%) cases. Thirty-eight (64.4%) cases were having peri-lesional or intra-lesional segments or involving a small segment enlargement of the pulmonary vessel. Among the cases showing DECT positivity, the typical pattern on the basis of the Radiological Society of North America (RSNA) classification was noted in 71.2% of cases, whereas the atypical pattern was found in 1.2% percent of cases and the intermediate type was depicted in 25.4% percent of cases. Forty-six point three percent (46.3%) of the total cases that were enrolled in the study were grouped as the no pneumonia category.

Conclusion

The result of this study proved that the maximum number of RT-PCR-positive COVID-19 patients had mild symptoms and few comorbidities with normal chest DECT and fell under the no pneumonia category of the RSNA CT classification of COVID patients. However, out of the remaining patients, the majority of patients had GGO on DECT as a typical finding mixed with other patterns in a bilateral distribution and peripheral predominance. A preponderance of patients presented with the typical appearance of pneumonia followed by an intermediate type.

## Introduction

Coronavirus disease (COVID-19) has been declared a pandemic and public health emergency of international concern since March 11, 2020 [1]. Severe acute respiratory syndrome coronavirus 2 (SARS COV 2, which is an encapsulated single-stranded ribonucleic acid (RNA) [1-2] emerged at the end of 2019 in Wuhan, a city in China. The clinical scenario of this disease has a broad spectrum, ranging from being asymptomatic to mild symptomatic to severely ill [1,3-4]. Image finding also depicts a wide spectrum and mainly includes ground-glass opacification with a basal and peripheral predominance as an initial manifestation to gradually transforming to consolidations [1,4-5] at later stages. The majority of COVID-19 sufferers have the complaint of disturbance of smell and taste, including dysgeusia, ageusia, hyposmia, and anosmia. A few also develop conjunctivitis and even have a positive viral polymerase chain reaction (PCR) in their conjunctival fluid. Cutaneous lesions can also develop, similar to any other viral infection; the most common one is an erythematous rash [5-6]. Dual-energy computed tomography (DECT) has been introduced so as to provide better imaging possibilities in reverse transcription (RT)-PCR positive COVID patients. DECT has an edge over CT in the creation of Iodine maps, which can be further used to quantify perfusion disorders within the lung parenchyma, e.g. in pulmonary hypertension or pulmonary embolism [7]. In this study, we aim to gain insight into the spectrum of various findings on color-coded, noncontrast DECT images in the general population so as to create a database that can help further study in this direction. We also aim to assess the spectrum of findings elicited on the different energy scale of noncontrast DECT, so as to decide whether it is beneficial for those patients who cannot be subjected to contrast DECT either due to allergy to iodine-based contrast or any other reason.

## Materials and methods

Patient cohort and study design

This is a monocentric, retrospective study conducted in our tertiary care center, which is a designated COVID-19 care center, between the months of May and July 2020. It has the facility of separate inpatient, quarantine, and intensive care units, and patients were admitted as per the patient’s health condition and severity of symptoms. Informed patient consent was waived by the institutional ethical committee, as no individual data were included. All the examinations were carried out in accordance with ethical standards, as enacted in the Declaration of Helsinki, 1964, and its amendments that occurred in due course of time. In this study, chest DECT was accomplished over symptomatic patients who were referred to our hospital from other district hospitals after being confirmed RT-PCR positive for SARS-COV-2. All symptomatic patients who presented with a variable degree of fever, cough, and dyspnea and fulfilled the inclusion, as well as exclusion criteria, were enrolled in this study.

Inclusion Criteria

Symptomatic patients with fever, cough, and dyspnea who are proven COVID-19 with positive RT-PCR and have undergone noncontrast DECT chest scans at a maximum of 28 days after the positive RT-PCR test.

Exclusion Criteria

All asymptomatic patients with positive RT-PCR-confirmed SARS-COV-2 infection.

On all the patients who fulfilled the above criteria, DECT scans were performed on the 384-slice dual-energy CT scanner (Somatom Force, Siemens Healthcare, Erlangen, Germany). After scout acquisition, imaging was conducted in a supine position and craniocaudal scanning direction. All the DECT images were acquired in a single shot where patients were asked to hold the breath in the inspiratory phase. Images were later reconstructed over the software using an increment of 0.7 mm into 1 mm slice thickness. As per guidelines, decontamination of the DECT suit, which was worn by the patient, was required and was performed using either 70% ethanol or 0.1% sodium hypochlorite as per the availability at the stance. After each DECT examination, there was passive air exchange allowed in the gantry area for a duration of 60 minutes.

Image analysis

Two radiologists (more than five years experience) reviewed the DECT images independently on an Osirix MD Workstation (Apple Mac; Apple Inc., Cupertino, California) [8]. Both the radiologists who were involved in the study were purposedly blinded to clinical data so as to avoid any bias. A detailed evaluation of the images was done and changes were identified, ranging from ground-glass opacity (GGO), consolidation, crazy-paving pattern, to various other parenchymal abnormalities. The location of lesions was assessed carefully and they were divided into different categories involving one lung (right or left) or both lungs. The number of lobes involved was calculated. Distribution of all the opacities in different zones of the bilateral lung field was done, and they were further classified as central, which is the inner two-third of the lung tissue, and peripheral, which extends as the outer one-third of the lung tissue. The distribution of lung abnormalities was further sub-segmented based on their location into anterior and posterior locations with respect to an imaginary line. This division is made as the parenchyma of the bilateral lung field anterior to a line drawn midway on axial DECT (imaginary line) was considered anterior and the remaining portion of the lung parenchyma was considered posterior. Lesions of lung parenchyma were also categorized into different entities using the Fleischner society glossary of terms for thoracic imaging [9]. GGO is established as an abnormal increase in lung density with non-obscuration of bronchial as well as vascular structures, whereas consolidation is explicated as lung parenchyma with increased density through which vascular and bronchial structures cannot be visualized. Furthermore, we evaluated for other associated abnormalities such as airway, vascular, pleural, and mediastinal abnormalities. A semi-quantitative scoring system has been used to quantitatively estimate the involvement of lung parenchyma by visually calculating the percentage of the total lung involved by dividing the right lung into three lobes and the left lung into two lobes [10].

Statistical analysis

The finding of this study was evaluated using the Statistical Package for Sciences (SPSS version 21.0; IBM Corp., Armonk, NY). In this, we have used categorical variables, which were specified as counts and percentages while continuous variables were directed as mean, range, and standard deviation. The conformity between two interpreting radiologists as required for DECT findings was conceived using the Kappa method. According to Landis and Koch, the following statistics are used: 0 elicited poor agreement; 0.01-0.20 showed slight agreement; 0.21-0.40 elicited fair agreement; 0.41-0.60 showed moderate agreement; 0.61-0.80 directs substantial agreement; whereas 0.81-1.0 depicts almost perfect agreement [1].

## Results

Demographics and clinical characterization with laboratory findings

A total of 110 patients were enrolled in the study, of which 80 were males and 30 were females with a mean age of 40.5 +/- seven years. (range 24-84 years). Out of 110 patients, 76 patients had a history of travel to high-risk zones within or outside the country or a history of close contact with a positive patient. Fever was the most frequently observed symptom seen in 60 patients followed by fatigue or malaise in 60 patients (54.5%), sore throat in 35 (31.8%) patients, and cough in 52 (47.3%) patients. Only 14 (12.7%) patients were dyspnoeic, 10 (9.1%) patients complained of tachypnea, and six (5.4%) patients had desaturation. Lymphopenia was documented in 50 patients (34%), whereas lymphocytosis was seen in 14 (9.5%). C-reactive protein was above the maximum limit of the standard normal range in 77 (52.4%). All these findings regarding patient demographics, clinical features, and laboratory investigations are summarized in Table 1.

**Table 1:** Demographic features, clinical features, and laboratory findings CLD: Chronic Liver Disease; WBC: White Blood Cells; CRP: C-Reactive Protein

Patient demographics	Number of patients (n=147)	Percentage
Mean age (years) ±S.D.	40.5 +/- 7	-
Gender		
Male	80	72.7
Female	30	27.3
History of contact with a Covid-19 patient or travel to a high-risk zone		
Present	76	69.1
Absent	14	12.7
Co-Morbid Illness		
Hypertension	7	6.3
Diabetes Mellitus	3	2.7
CLD	2	1.8
Rheumatoid Arthritis	0	0
Clinical Features		
Fever	60	54.5
Cough	52	47.3
Sore Throat	35	31.8
Dyspnea	14	12.7
Malaise/Fatigue	60	54.5
Increased Respiratory Rate (> 30/min)	10	9.0
Reduced Oxygen Saturation (< 90%)	6	5.4
Lab Investigations		
Lymphocyte count (Normal value 1.1 - 3 x 10^9^ / L)		
Increased	14	9.5
Decreased	50	34
Increased CRP (normal value < 10 mg/L)	77	52.4

Chest DECT findings

**Figure 1:**
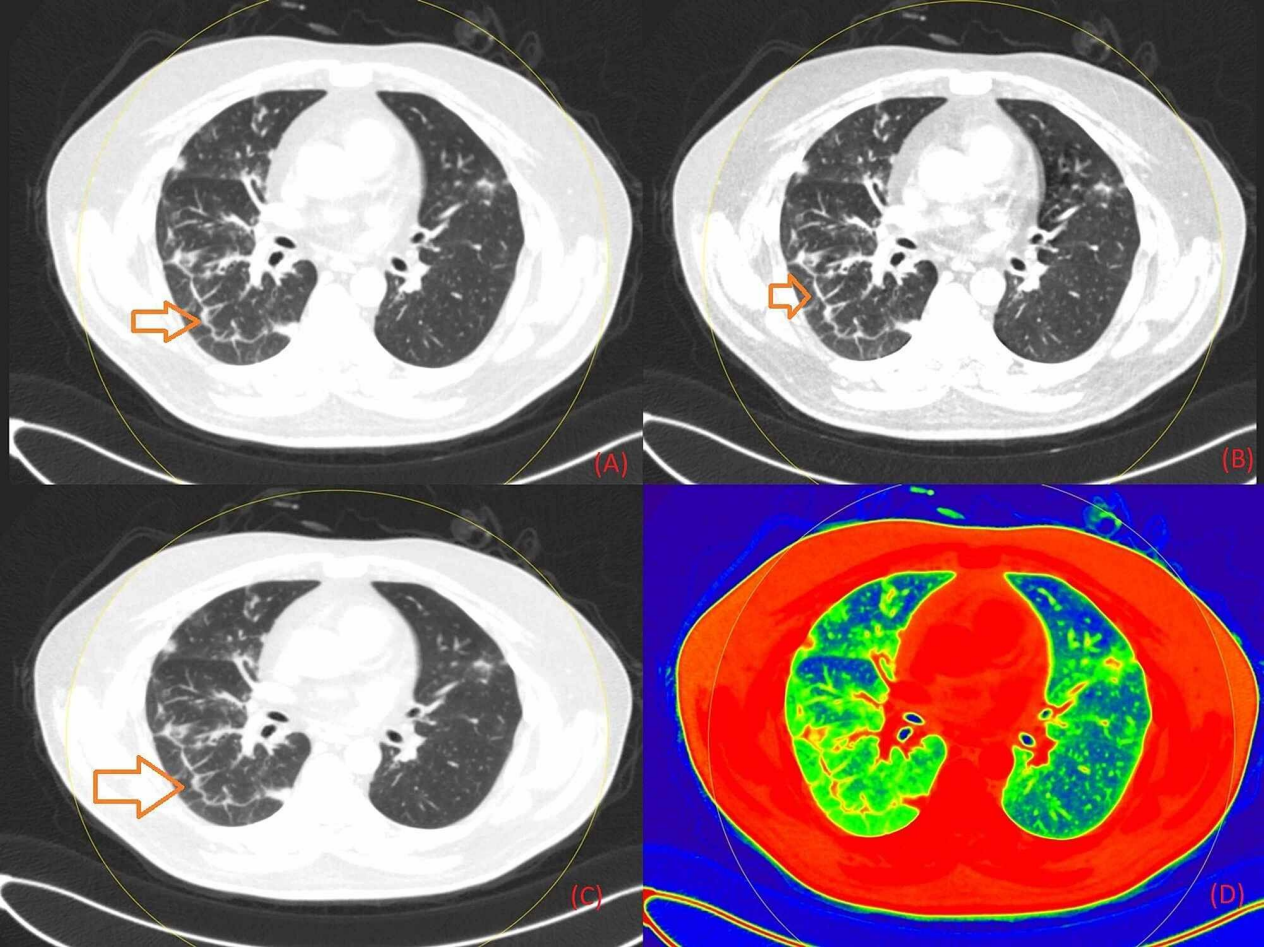
Non-contrast chest DECT axial images representing fibrosis interspersed with GGO in the lower lobe of the right lung field (marked with red arrows) in grayscale (Figure 1A), on 40 keV energy (Figure 1B), on 190 keV energy (Figure 1C), and on color-coded presentation on basis of atomic number (Figure 1D) DECT: Dual-Energy Computed Tomography; GGO: Ground-Glass Opacity

**Figure 2:**
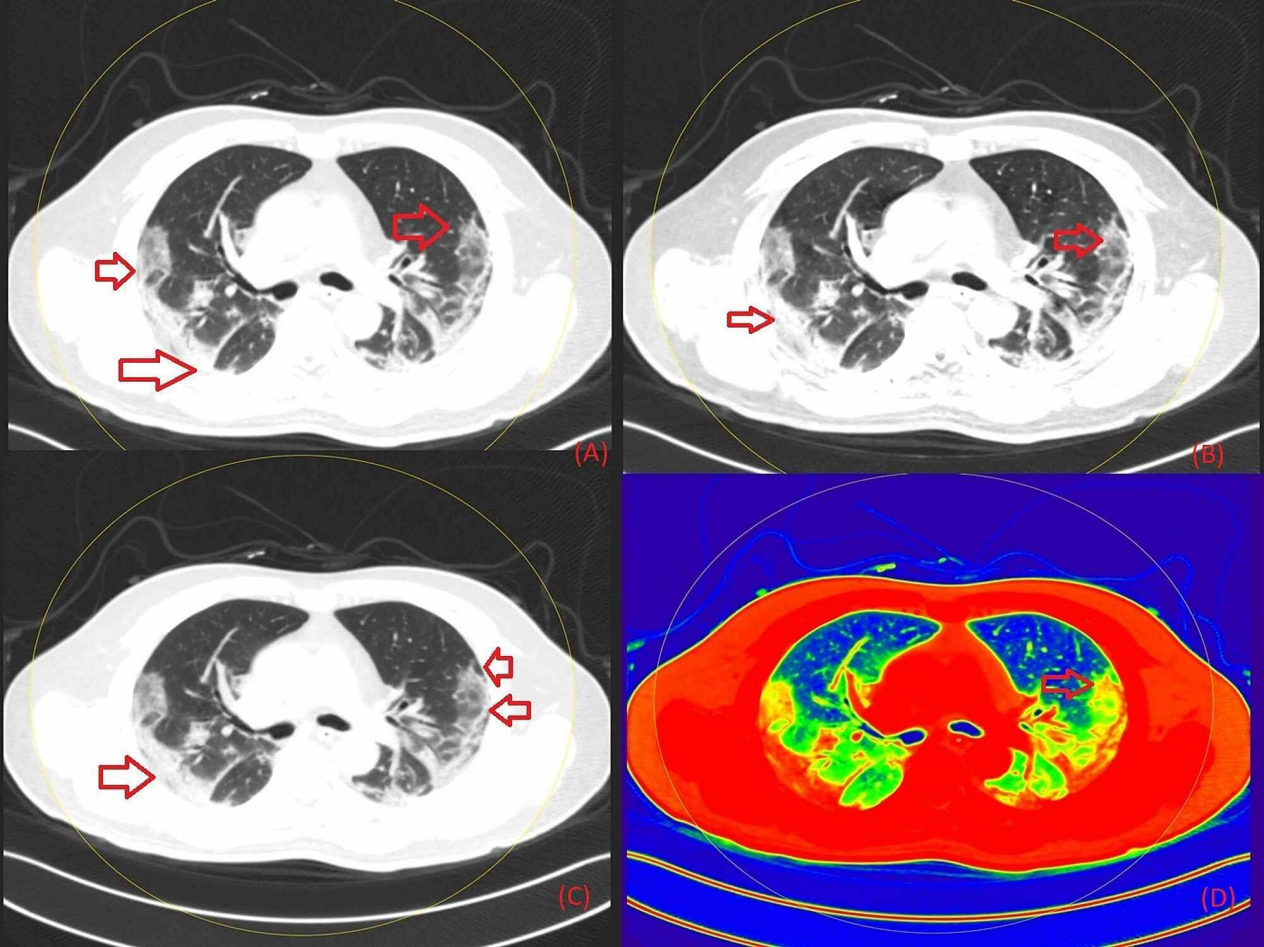
Non-contrast chest DECT axial images representing diffuse GGO (marked with red arrows) in the bilateral lung field predominantly in peripheral distribution, where Figure 2A is the section in grayscale, Figure 2B in 40 keV energy, Figure 2C in 190 keV energy, and Figure 2D as a color-coded image based on atomic number DECT: Dual-Energy Computed Tomography; GGO: Ground-Glass Opacity

**Figure 3:**
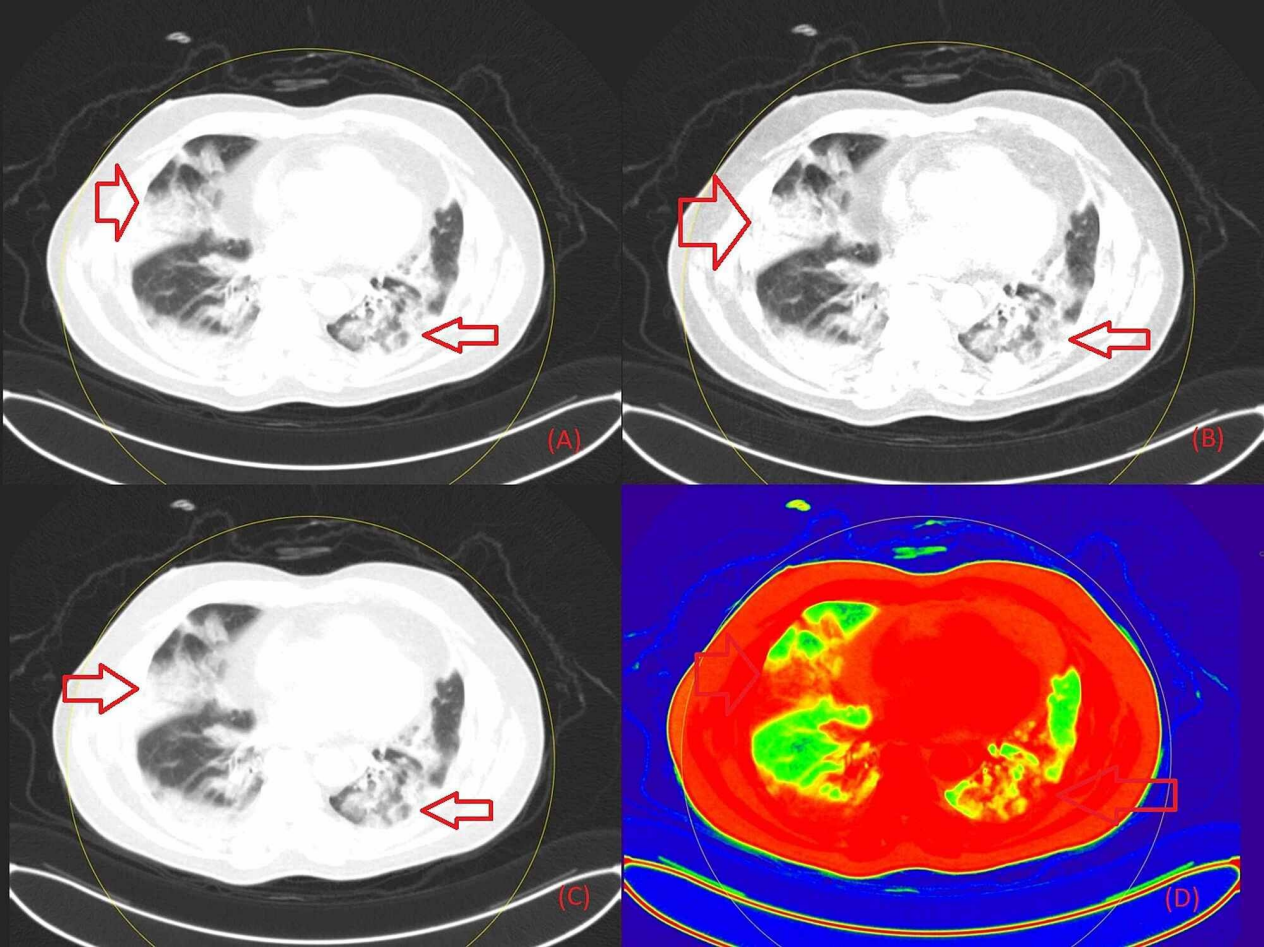
Non-contrast chest DECT axial images in a 60-year-old COVID-19 RT-PCR positive patient showing consolidation with an air bronchogram in the upper lobe of the right lung field and the lower lobe of the left lung field (red arrows) Figure 3A is the section in grayscale, Figure 3B in 40 keV energy, Figure 3C in 190 keV, Figure 3D as a color-coded image based on atomic number. DECT: Dual-Energy Computed Tomography; RT-PCR: Reverse Transcription-Polymerase Chain Reaction

**Figure 4:**
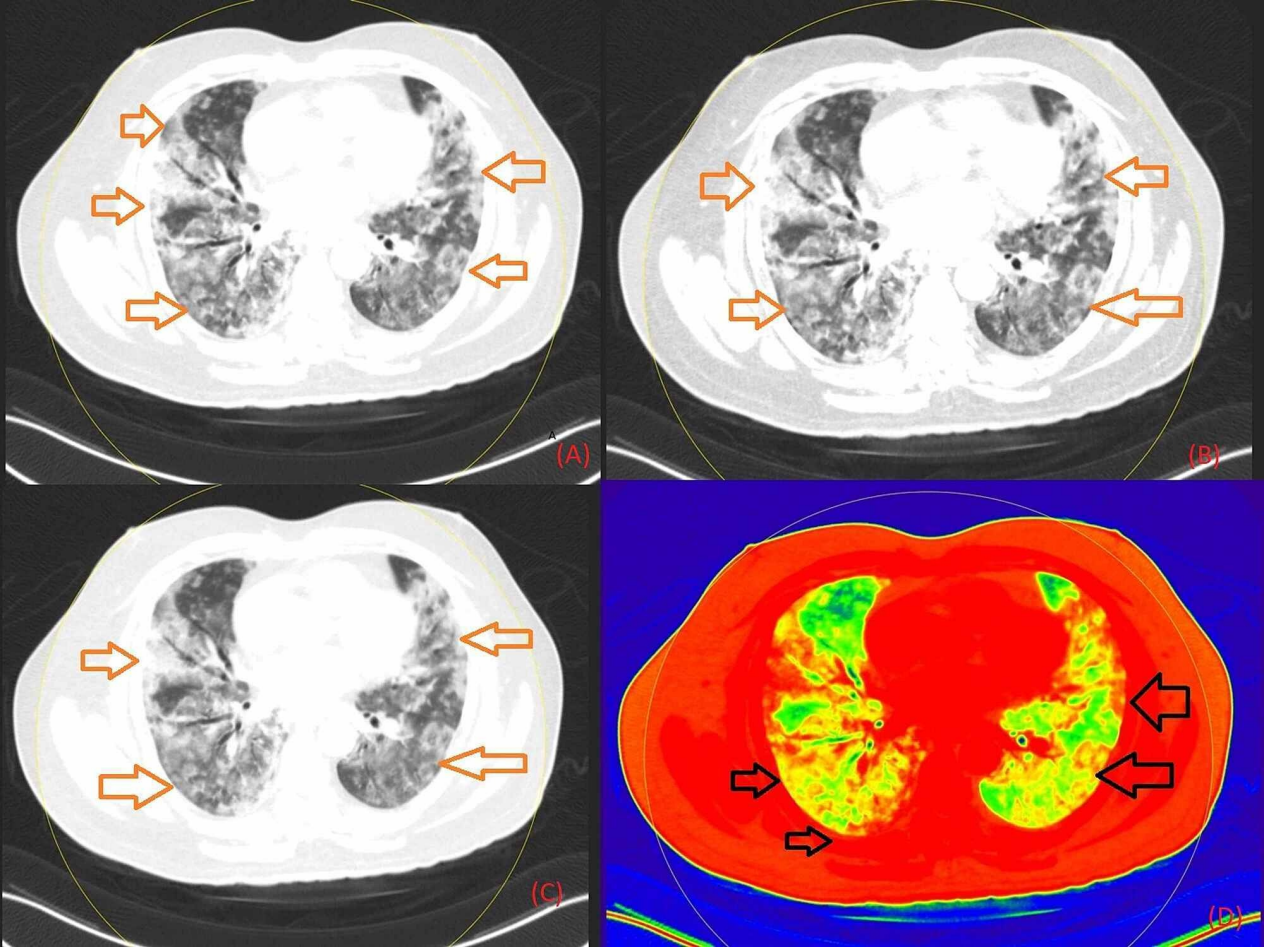
Non-contrast axial chest DECT images in the lung window setting of a COVID-19 RT-PCR-positive patient showing bilateral, elongated, confluent GGO with pronounced peripheral and posterior distribution (marked with red arrows) with interlobular septal thickening producing a crazy-paving pattern with tractional bronchiectasis, which can be appreciated on grayscale (Figure 4A), on image setting of 40 keV (Figure 4B), of 190 keV (Figure 4C), and a color-coded image (Figure 4D). DECT: Dual-Energy Computed Tomography; GGO: Ground-Glass Opacity; RT-PCR: Reverse Transcription-Polymerase Chain Reaction

**Figure 5:**
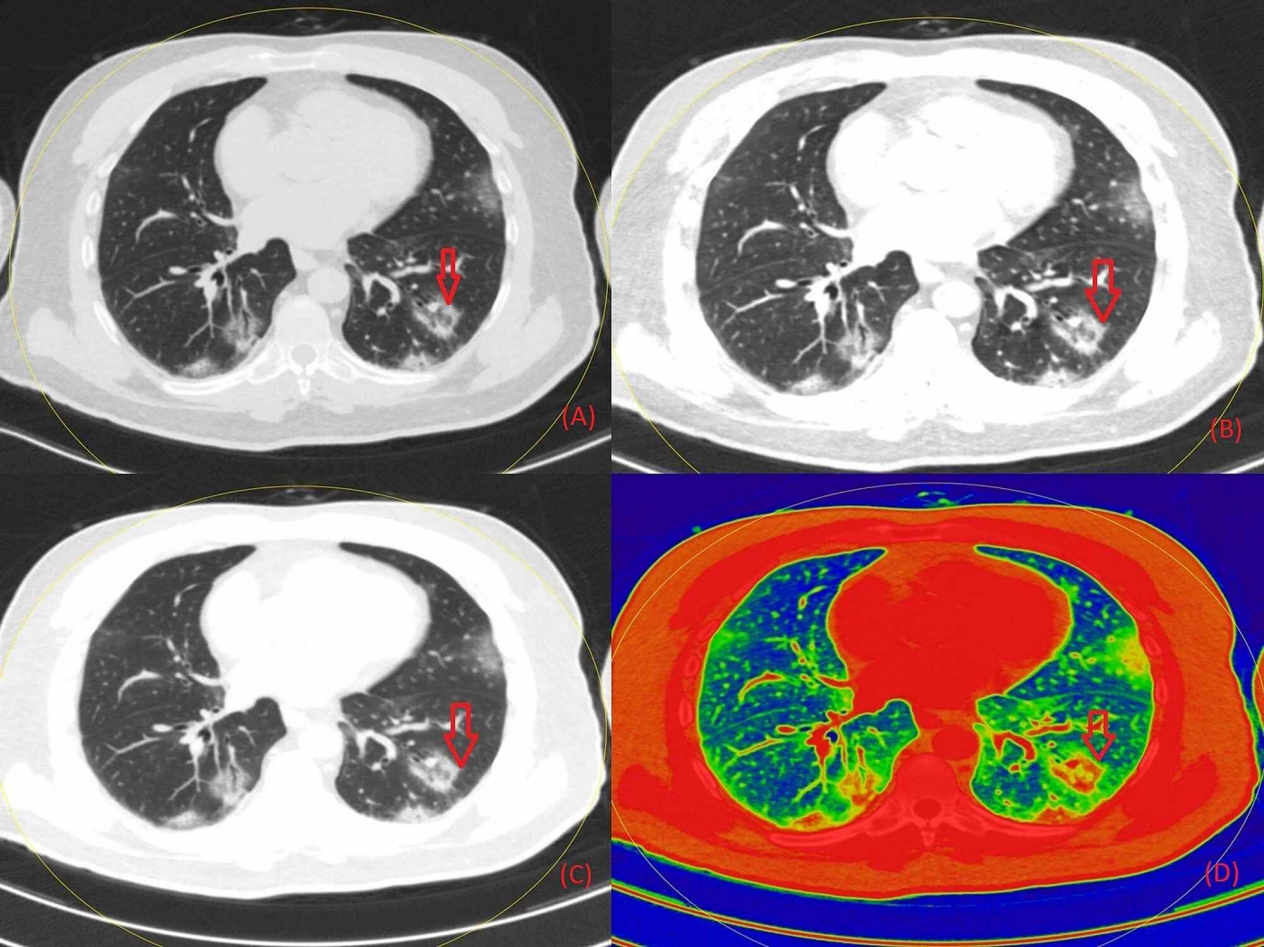
Non-contrast axial chest DECT images in the lung window setting of a 40-year-old, male, COVID-19 RT-PCR-positive patient showing central GGO surrounded by consolidation in a crescent shape (marked with red arrows), also known as reverse halo sign or atoll sign on a grayscale image (Figure 5A) This finding can also be appreciated on a lung window of 40 keV energy (Figure 5B) of 190 keV energy (Figure 5C ) and on color-coded depiction (Figure 5D). DECT: Dual-Energy Computed Tomography; GGO: Ground-Glass Opacity; RT-PCR: Reverse Transcription-Polymerase Chain Reaction

**Figure 6:**
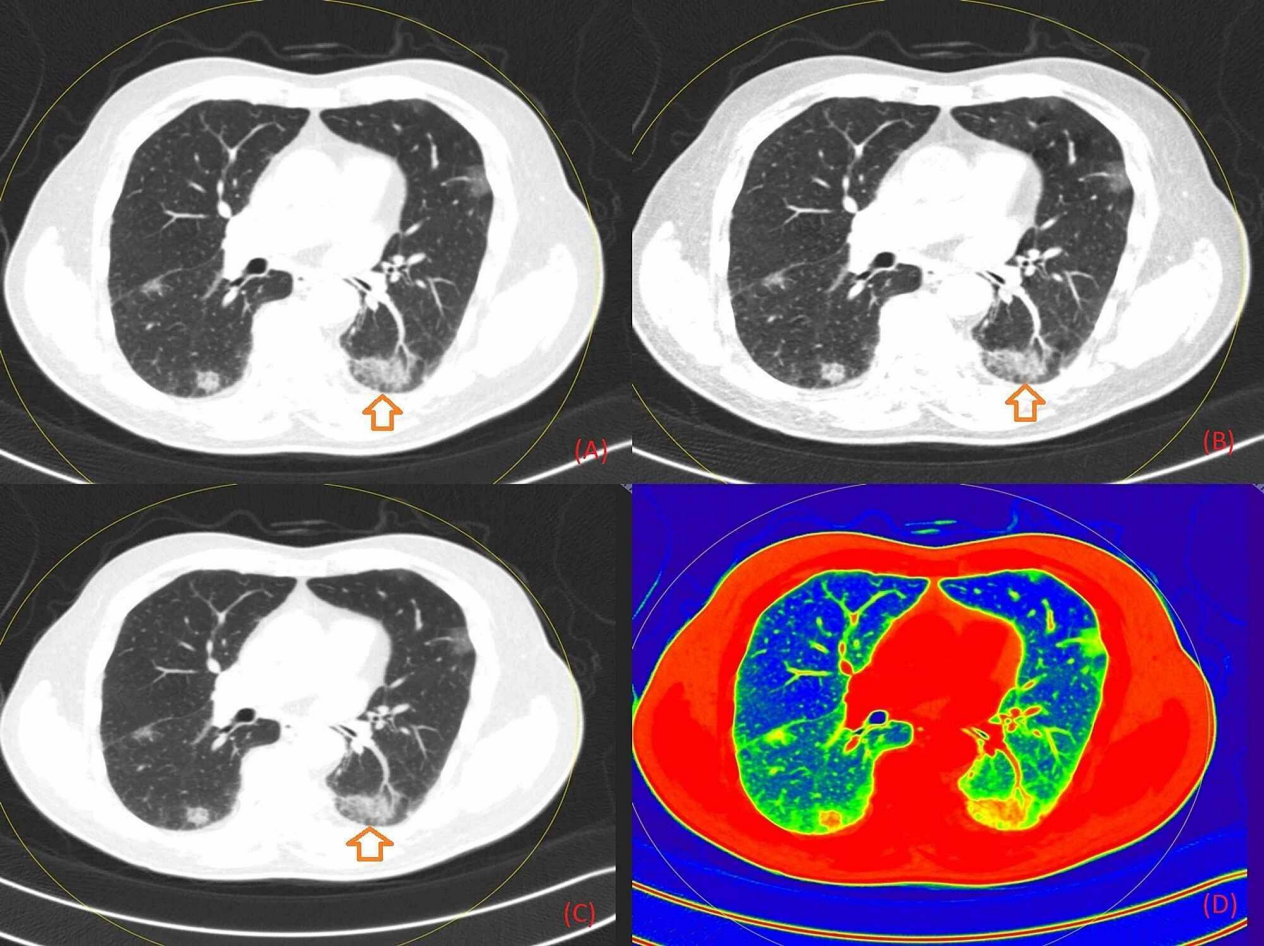
An axial non-contrast chest DECT image obtained in a 42-year-old male shows patchy GGO in the lower basal segment of the right and left (marked with arrows) lung fields Figure 6A is the section in grayscale, Figure 6B in 40 keV energy, Figure 6C in 190 keV energy, and Figure 6D as a color-coded image based on atomic number. DECT: Dual-Energy Computed Tomography; GGO: Ground-Glass Opacity

It was observed that there was almost perfect agreement (Cohen’s Kappa of 0.83) in reading noncontrast DECT images between the two primary radiologists. Lung parenchyma showed a wide spectrum of changes in RT-PCR-positive cases, from being completely normal to showing various findings in multiple lobes of bilateral lung fields (Figure 1). Multiple lobe involvement was seen more frequently (see Figures 1-6). Twenty-six (44.1%) had involvement of all five lobes, whereas two-lobe and single lobe involvement was seen in seven (11.8%) patients each. In axial sections, we observed distribution in peripheral areas is the most common, seen in 57 (96%) cases, among which 40 (67.8%) had solely peripheral distribution, whereas 17 (28.8%) has peripheral as well as central distribution and neither of the patients showed purely central distribution. In consideration of the anterior-posterior sections, 0% of cases have shown solely anterior distribution, whereas 29 (49.2%) cases have shown solely posterior distribution, whereas 26 (44.1%) cases have shown combined anterior and posterior distribution. Reticulations were seen in 17 (28.8%) cases, whereas pure GGO (Figure 2) was observed in 16 (28%). A crazy-paving pattern (Figure 4) is produced when GGO combines with interlobular septal thickening and intralobular lines. A relatively less number of cases showed subpleural curvilinear lines, i.e. in 11 patients (18.6%). The reverse halo sign (Figure 5), also known as the atoll sign, was seen in 11 (18.6%) and the air bronchogram sign (Figure 3) was seen in 14 (23.7%) cases, respectively. Eight patients (13.6%) show bronchial wall thickening, whereas three patients (5.1%) show bronchodilatation. None of the patients showed the halo sign, cavitation, pleural effusion, or mediastinal lymphadenopathy. Patchy GGO is also observed in a few patients (Figure 6). These findings are cumulated below in the form of Table 2 and Table 3.

**Table 2:** Distribution of lung findings on chest DECT DECT: Dual-Energy Computed Tomography

Lung parenchymal abnormalities on DECT	No. of patients	Percentage
Present	59	53.6
Absent	51	46.3
Laterality of Lung Involvement		
Bilateral	43	72.8
Right Lung	7	11.8
Left Lung	8	13.6
Lobar Involvement		
Right Upper Lobe	50	84.74
Right Middle Lobe	32	54.2
Right Lower Lobe	41	69.5
Left Upper Lobe	44	74.6
Left Lower Lobe	42	71.2
Number of Lobes Involved		
5 Lobes	26	44.1
4 Lobes	14	23.7
3 Lobes	4	6.7
2 Lobes	7	11.8
1 Lobe	8	13.6
Axial Location of Opacity		
Central (inner 2/3^rd^ of Lung)	0	0
Peripheral (outer 1/3^rd^ of Lung)	40	67.8
Central and Peripheral	17	28.8
Antero-Posterior Location		
Anterior	0	0
Posterior	29	49.1
Anterior and Posterior	26	44.1

**Table 3:** Type of lung opacities and additional findings on chest DECT DECT: Dual-Energy Computed Tomography; GGO: Ground-Glass Opacity

Lung opacity	No. of patients	Percentage
GGO	59	100
Pure GGO	16	28
GGO with Crazy Paving Pattern	17	28.8
Pure Consolidation	0	0
Mixed Pattern (GGO with Consolidation)	25	42.4
Subpleural Linear / Curvilinear Lines	11	18
Nodules	3	5
Reticulations	17	28.8
Halo Sign	0	0
Reverse Halo Sign	11	18.6
Cavitation	0	0
Perilesional / Intralesional Vessel Enlargement	38	64.4
Bronchial Wall Thickening	8	13.6
Bronchial Dilatation	3	5.9
Air Bronchogram Sign	14	23.7
Air Bubble sign	3	5.1
Additional Findings		
Pleural Effusion	0	0
Pericardial Effusion	0	0
Mediastinal Lymphadenopathy	0	0

## Discussion

The Radiological Society of North America (RSNA) has recently released a consensus statement, which has been endorsed by the Society of Thoracic Radiology and the American College of Radiology, which has classified the various CT appearances of COVID-19 into four major categories, so as to establish a standardized reporting language that can be used by any doctor in any part of the world. This will ease the sharing of information among different parts of the world, and more conclusive data can be accumulated [11]. It has four major groups as typical appearance, intermediate appearance, atypical appearance, and negative for pneumonia. Typical appearance involves peripheral bilateral GGO with the presence or absence of consolidation. The atoll sign also signified the typical appearance. In our study, 42 (71.2%) cases showed typical appearance [7]. The intermediate appearance had a major absence of typical findings, and the presence of diffuse, multifocal, perihilar, or unilateral GGO, with or without consolidation, lacking a specific distribution, and are non-rounded or non-peripheral were present in 15 cases (25.4%) [7]. In our study, two (1.2%) cases showed the atypical appearance, which covers the absence of both typical and intermediate appearance with a predominant presence of isolated lobar or segmental consolidation without GGO, along with the presence of small nodules in discrete distribution and lung cavitation. The negative for pneumonia finding is given to patients where the CT chest is devoid of any finding that is suggestive of pneumonia, which is seen in 51 (46.3%) cases. In other words, there is in particular absence of GGO or consolidation [7]. We have conducted this retrospective study at our COVID-19 care center (CCC) to gain a wider view of the chest on non-contrast DECT of COVID-19 RT-PCR-positive patients in our population so as to gather more information about the various spectrums of COVID findings. Li K et al. conducted a study with positive COVID-19 cases that presented with a wide range of clinical symptoms and found that 71.8% had a CT positivity rate, of which 30.8% were mild cases, 59% common, and 10.2% severe cases, respectively [12]. Caruso D et al. observed various pulmonary findings in 96.6% of symptomatic COVID-positive patients on chest CT [13]. Yu M et al. reported a CT positivity rate of 100% in their cohort study [14]. In our study, we have concluded that among COVID RT-PCR-positive patients who have lung parenchymal abnormalities, bilateral and multilobar distribution of pulmonary opacities with a peripheral predilection was observed. Our study has been successful in collecting data that fairly collaborate the distribution and type of pulmonary opacities observed in various COVID-positive patients. These patterns are cumulated in Table 4.

**Table 4:** Different pattern of COVID-19 pneumonia classified by the RSNA chest CT classification system CT: Computed Tomography: RSNA: Radiological Society of North America

Type of pattern	Number of patients presented	Percentage
Typical pattern	42	71.2%
Intermediate pattern	15	25.4%
Atypical pattern	2	1.2 %

The most dominant abnormality of lung parenchyma observed in the maximum cases is GGO, in the form of pure GGO (100%), GGO superimposed with consolidation (42.4%), and GGO with admixed crazy-paving pattern (28.8%). These findings are in concordance with multiple studies conducted in different parts of the world and summarized in the systemic review by Saheli et al., wherein it was found that GGO was a dominant lung parenchymal abnormality found in 88% of cases across 22 studies reported from various countries. The typical pattern was observed in 71.2% of cases, whereas atypical was found in 1.2% of cases. The intermediate type was depicted in 25.4% of cases, whereas 46.3% of total cases showed no pneumonia sign. Our findings are consistent with Caruso D et al. who observed enlargement in vessel size in 89% of patients and Yan Li et al. who observed vascular enlargement in 82.4% [13-14]. Bai HX et al. observed that enlargement in vessel size has been frequently associated with COVID-19 pneumonia in comparison to non-COVID-19 pneumonia with a significant pP-value (<0.001) [15]. In severe cases, the percentage of diseased lung parenchyma was relatively higher than the cases with a mild form of the disease.

## Conclusions

In conclusion, we found a high proportion of cases of normal chest DECT in mildly symptomatic, laboratory-confirmed SARS-CoV-2 patients, which fell under the no pneumonia category of the RSNA CT Classification system. Patients with a positive DECT series elicited a predominance of bilateral and multilobar distributions of GGO with a posterior and peripheral predilection, which was similar to the findings reported in other studies. The majority of patients who were DECT positive presented with typical appearances of pneumonia followed by the intermediate type.
